# Cellular Chaperones As Therapeutic Targets in ALS to Restore Protein Homeostasis and Improve Cellular Function

**DOI:** 10.3389/fnmol.2017.00251

**Published:** 2017-09-08

**Authors:** Bernadett Kalmar, Linda Greensmith

**Affiliations:** ^1^Sobell Department of Motor Neuroscience and Movement Disorders, UCL Institute of Neurology London, United Kingdom; ^2^MRC Centre for Neuromuscular Disease, UCL Institute of Neurology London, United Kingdom

**Keywords:** protein misfolding, chaperones, heat shock protein, amyotrophic lateral sclerosis, stress response

## Abstract

Heat shock proteins (Hsps) are ubiquitously expressed chaperone proteins that enable cells to cope with environmental stresses that cause misfolding and denaturation of proteins. With aging this protein quality control machinery becomes less effective, reducing the ability of cells to cope with damaging environmental stresses and disease-causing mutations. In neurodegenerative disorders such as Amyotrophic Lateral Sclerosis (ALS), such mutations are known to result in protein misfolding, which in turn results in the formation of intracellular aggregates cellular dysfunction and eventual neuronal death. The exact cellular pathology of ALS and other neurodegenerative diseases has been elusive and thus, hindering the development of effective therapies. However, a common scheme has emerged across these “protein misfolding” disorders, in that the mechanism of disease involves one or more aspects of proteostasis; from DNA transcription, RNA translation, to protein folding, transport and degradation via proteosomal and autophagic pathways. Interestingly, members of the Hsp family are involved in each of these steps facilitating normal protein folding, regulating the rate of protein synthesis and degradation. In this short review we summarize the evidence that suggests that ALS is a disease of protein dyshomeostasis in which Hsps may play a key role. Overwhelming evidence now indicates that enabling protein homeostasis to cope with disease-causing mutations might be a successful therapeutic strategy in ALS, as well as other neurodegenerative diseases. Novel small molecule co-inducers of Hsps appear to be able to achieve this aim. Arimoclomol, a hydroxylamine derivative, has shown promising results in cellular and animal models of ALS, as well as other protein misfolding diseases such as Inclusion Body Myositis (IBM). Initial clinical investigations of Arimoclomol have shown promising results. Therefore, it is possible that the long series of unsuccessful clinical trials for ALS may soon be reversed, as optimal targeting of proteostasis in ALS may now be possible, and may deliver clinical benefit to patients.

## Introduction

Amyotrophic Lateral Sclerosis is a fatal neurodegenerative disease affecting motor neurons, which results in muscle wasting, paralysis and death, typically within 2–5 years of diagnosis. The disease has a prevalence of 4–6 per 100,000 population, with a lifetime risk of 1:600 (Al-Chalabi et al., 2016). Similar to other neurodegenerative diseases, ALS affects specific neuronal populations, in the case of ALS, upper motor neurons in the motor cortex and lower motor neurons in the spinal cord. The resulting motor neuron dysfunction and death leads to a progressive loss of voluntary muscle function and ultimately death, usually from respiratory failure.

Although our understanding of ALS pathomechanisms has advanced significantly in recent decades, in part due to the discoveries of over 50 ALS-causing genes and the generation of animal models recapitulating the disease (Taylor et al., 2016), there is still no effective disease modifying therapy that has a significant impact on patient outcomes. This is likely due to the highly complex nature of ALS, which involves deficits not only in motor neurons but also non-neuronal cells including glial, muscles and even the immune system. Furthermore, ALS pathology not only involves multiple cell types but it also affects multiple cellular processes, including axonal transport, endosomal trafficking, autophagy, proteasomal degradation, calcium mishandling and mitochondrial function. However, a common theme that links many of the processes involved in ALS pathogenesis is that they are potentially caused by the abnormal maintenance of the proteins that are involved in these functions, resulting in their misfolding and/or aggregation. Thus, it is possible that ALS is a protein misfolding disease, in which deficits in the various stages of protein homeostasis, including RNA synthesis, processing, protein translation, folding and degradation can result in the diverse range of cellular deficits that are known to play a role in ALS. Therefore, the development of strategies that target specific and individual disease pathways is unlikely to be successful in modifying disease progression in ALS, or may, at best, be only partially effective, as other elements of pathology remain unaltered. However, improving protein homeostasis and strengthening protein quality control mechanisms could be an effective complimentary approach to targeting specific pathways.

One such attractive target for drug development in ALS is the HSR, an intrinsic cellular defense mechanism that can be targeted to combat protein dyshomeostasis, which in turn may prevent or ameliorate the development of a whole range of secondary functional deficits. In this review we summarize the evidence that shows that targeting the HSR, by upregulating a core set of heat shock proteins (Hsps), may be an effective therapeutic strategy for ALS.

## ALS is a Protein Misfolding Disease

**FIGURE 1:**
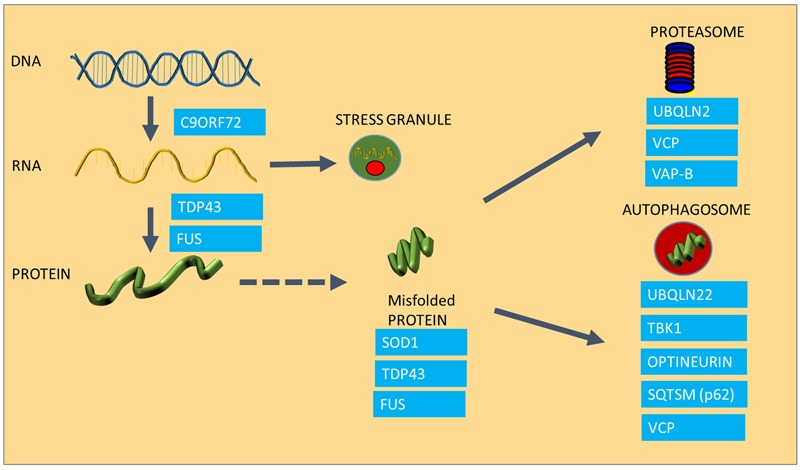
The involvement of ALS causing mutations in protein homeostasis. The Figure summarizes the areas of protein homeostasis that are perturbed in ALS, and the relevant disease-causing mutations that are linked to protein quality control. Synthesis of mRNA species is disturbed in mutants expressing hexanuceotide repeat expansions in the C9ORF72 gene, resulting in the generation of abnormal RNA species. FUS and TDP-43 control mRNA processing and defects in this process result in abnormal stress granule formation under stress and reduced expression and aggregation of TDP-43. Some ALS causing proteins are highly aggregation prone, such as SOD1, TDP-43 and FUS. Mutations in UBQLN2, VCP and VAP-B are linked to proteasomal dysfunction, whereas UBQLN, TBK1, Optineurin, Sequestrosome and VCP are involved in autophagy, which is also impaired in ALS.

One of the most characteristic neuropathological features of ALS is the presence of cytoplasmic inclusions in degenerating motor neurons in post-mortem tissues of ALS patients (Ince et al., 1998; Okado-Matsumoto and Fridovich, 2002; Shaw et al., 2008), and is indicative of disturbances in protein homeostasis, or proteostasis. The presence of ubiquitinated inclusions containing components of the cytoskeleton, various elements of the protein maintenance machinery, such as TDP-43 and Hsps are characteristic for both familial and sporadic ALS cases (Watanabe et al., 2001; Arai et al., 2006; Neumann et al., 2006; Pokrishevsky et al., 2012). The presence of ubiquitin in these aggregates implies that the proteins that have been sequestered into aggregates were marked for degradation and for some reason, the cell was not able to degrade them (Garofalo et al., 1991; Leigh et al., 1991). Recent advances in understanding of the genetic causes of ALS have revealed that a wide range of the genes that are implicated in the disease encode proteins that play roles in different stages of protein homeostasis, including protein synthesis, folding and clearance, and many of these mutated proteins have been found to be aggregated in ALS patient tissues and also in animal models of ALS (see **Figure 1** for a summary of ALS genes in relation to protein homeostasis). The first ALS-causing mutation discovered was the mutation in the SOD1 gene (Rosen et al., 1993). Although the protein itself does not play a role in the maintenance of other proteins, mutant SOD1 protein forms aggregates with other proteins as well as cellular organelles such as mitochondria (Pasinelli et al., 2004; Ahtoniemi et al., 2008; Li et al., 2010). Since the discovery of mutant SOD1, all other ALS causing mutations had been implicated in either protein synthesis or degradation pathways (**Figure 1**). The discovery of TDP-43 and FUS mutations in ALS (Sreedharan et al., 2008; Kwiatkowski et al., 2009) established a link between ALS and RNA processing and transport, a crucial regulatory process in protein translation. ALS-causing mutant TDP-43 and FUS, which play crucial roles in the normal processing of long mRNA, are also major components of the protein aggregates observed in motor neurons of familial forms of ALS (except SOD1-ALS), as well as sporadic cases of ALS in the absence of TDP-43 and FUS mutations (Arai et al., 2006; Neumann et al., 2006). TDP-43 and FUS thus have an important role in regulating the level of expression of huge number of proteins through regulation of mRNA processing (Lagier-tourenne et al., 2012). TDP-43 and FUS proteins possess aggregation-prone prion domains. Due to these prion domains TDP-43 and FUS mutants are even more susceptible to aggregation (Patel et al., 2015). Interestingly, TDP-43 function and clearance is regulated by heat shock and the transcription factor for the HSR, HSF-1 (Chen et al., 2016; Lin et al., 2016; Li et al., 2017).

An abnormally long intronic hexanucleotide repeat in the C9ORF72 gene is now known to be a major cause of ALS, accounting for 40% of familial cases and 8% of sporadic ALS (DeJesus-Hernandez et al., 2011; Lagier-tourenne et al., 2012). It is currently thought that the hexanucleotide repeat intronic sections of the gene are transcribed in both directions of the gene, causing the appearance of RNA species accumulating into RNA foci, which are essentially RNA containing inclusions (DeJesus-Hernandez et al., 2011; Taylor et al., 2016), which are crucial in maintaining a balance in the amount and quality of synthesized proteins. Under conditions of cellular stress, crucial mRNA and protein components of the cell are assembled into stress granules in order to preserve these species and to ensure that protein synthesis can be restored rapidly after the stress conditions have resolved. However, in cells expressing ALS-causing TDP-43 mutations, disaggregation of stress granules is delayed following oxidative stress and TDP-43-containing stress granules persist (Parker et al., 2012; Fan and Leung, 2016). C9ORF72 mutant cells appear to undergo transcription, resulting in the production of abnormal RNA and dipeptide repeat products that in turn get deposited in RNA foci (RNA species) or in protein aggregates (dipeptides products of abnormally translated hexanucleotide repeats), causing toxicity as well as acting as sinkholes for other functional proteins (Lagier-tourenne et al., 2012; Mizielinska et al., 2014).

The widespread appearance of protein aggregates in ALS implies that a generalized protein misfolding phenomenon may underlie disease, affecting both mutated and normal bystander proteins which become caught up in aggregates, resulting in a depletion of functional proteins such as chaperoning Hsps, the very agents in the cell which are responsible for maintaining correct protein folding (Ince et al., 1998; Watanabe et al., 2001). The high ubiquitin content in ALS aggregates also suggests that cells have recognized the misfolded proteins and have attempted to clear these through either the proteasome or through autophagy, both of which are impaired in ALS (Kabashi et al., 2004, 2012; Li et al., 2008; Cheroni et al., 2009; Chen et al., 2012). In addition, protein misfolding and the accumulation of misfolded proteinaceous species can elicit a protective response from the ER, the Unfolded Protein Response (UPR), which, if it persists, can turn into a self-destructive cascade leading to cell death and exacerbation of the disease (Kikuchi et al., 2006; Atkin et al., 2008). It has been shown that the most vulnerable fast motor neurons in the spinal cord are more prone to activate this ER stress pathway in ALS, and this may be the underlying cause of their heightened vulnerability (Saxena et al., 2009).

Protein aggregation is not only the result of a disturbance in protein folding, but it is also the result of a failure of the two major degradation pathways, the ubiquitin proteasome system (UPS) and the autophagy machinery, which also contribute to accumulation of misfolded species (Shahheydari et al., 2017). In the case of ALS, several disease-causing mutations have a functional role in protein degradation (**Figure 1**). For example Ubiquilin2 (UBQLN2) and Sequestosome-1 (SQSTM1) are adaptors in both the proteasome and autophagy pathways (Gal et al., 2009; Osaka et al., 2016), whereas Optineurin (OPTN) is an autophagy receptor, mediating the clearance of dysfunctional mitochondria through phosphorylation of another disease causing gene product, TBK-1 (Wong and Holzbaur, 2014; Moore and Holzbaur, 2016; Richter et al., 2016). The Valosin Containing Protein (VCP), and Vesicle Associated Protein B (VAP B), which have been found mutated in small number of ALS cases, play a role in the clearance of misfolded proteins through the ER (ER associated degradation; ERAD) as well as proteasomal sorting of ubiquitinated proteins (Kuijpers et al., 2013; Meyer and Weihl, 2014; Moustaqim-Barrette et al., 2014).

Thus, we now understand that protein maintenance is perturbed at multiple levels in ALS and most likely this manifests at a functional level in dysfunction of a myriad of cellular processes, including mitochondrial dysfunction, ROS production, axonal transport deficits, activation of the inflammatory pathway as well as the apoptotic pathway, not only in motor neurons but also in non-neuronal cells.

## The Relevance of the Heat Shock Response to Normal Protein Homeostasis

Since aggregation of proteins and RNA species is a characteristic feature of ALS, the development of therapies that aim to reduce protein aggregation has been investigated widely as it likely to have a significant impact in this disease. The HSR is a naturally occurring, endogenous cytoprotective pathway that exists in all cells, which acts to maintain protein homeostasis. A large, specialized family of proteins called Hsps exist to execute the HSR by chaperoning and folding client protein substrates and to monitor and ensure protein quality control. Naturally, chaperone systems are required under normal conditions for the maintenance of intracellular protein systems, and play a role in all aspects of the proteostasis network, including (i) protein synthesis, when chaperones are responsible for keeping polypeptide chains in a folding competent state; (ii) in the transport of synthesized proteins to their appropriate cellular destination; and (iii) in the correct folding of transported proteins upon arrival at their destinations. Ultimately chaperones ensure that functionally active enzymes and proteins are in their correct location at the right time and in the correct conformation to undertake their cellular function. Each crucial cellular compartment, the cytosol, ER and mitochondria, all have a specialized set of Hsps.

Hsps are a large family of chaperone proteins that are classified according to their molecular weight (small Hsps, Hsp40, Hsp60, Hsp70, Hsp90, and Hsp104) and in the latest nomenclature they are named after the gene that encodes for them (Kalmar and Greensmith, 2008; Radons, 2016). One of the most researched Hsp for regulatory and chaperone activity is the 70 kDa Hsp70, which is coded by 17 genes in the mammalian genome and has isoforms in the cytosol (HspA1 or Hsp70), the ER (HspA5, also called BiP), and in mitochondria (HspA9, also called mortalin or mt Hsp70). In the cytosol this Hsp has constitutive (Hsp72 or HspA8) and inducible isoforms (Hsp73 or HspA1). The Hsp70 protein has a large number of interacting partners, so called co-chaperones that aid its function. Co-chaperones of Hsp70 affect either effect substrate specificity of the complex or the kinetics of the folding cycle- this latter is regulated by nucleotide exchange factor co-chaperones (Young, 2010; Bracher and Verghese, 2015). Together, these chaperone proteins make up 10% of the total protein pool in the cytosol, indicating their importance in keeping functional protein systems in place (Finka and Goloubinoff, 2013).

Under normal conditions, cytosolic Hsp70, HspA1 (inducible) and HspA8 (constitutive), regulates the rate of translation at the ribosome, through folding of nascent proteins during synthesis (Horton et al., 2001; Zimmer et al., 2001). The other large Hsp family are members of the Hsp90 family, which form protein complexes with Hsp70. These larger molecular weight ATPase Hsp90 chaperones also have a specialized role in self-regulating cellular chaperones levels by binding to the master regulator of Hsp expression, the transcription factor HSF-1. Upon stress, when Hsp90 is recruited to chaperone large globular proteins, HSF-1 is released and is able to trimerize, enter the nucleus and initiate the transcription of heat shock responsive genes (Kalmar and Greensmith, 2008). Hsp90 has a crucial role recognizing large and intrinsically instable proteins, aiding their folding (Taipale et al., 2010). These client proteins are usually large receptor and enzyme complexes, among others steroid receptors, protein kinases and the assembly of small ribonucleoproteins as well as RNA polimerases (Buchner and Li, 2013).

**FIGURE 2:**
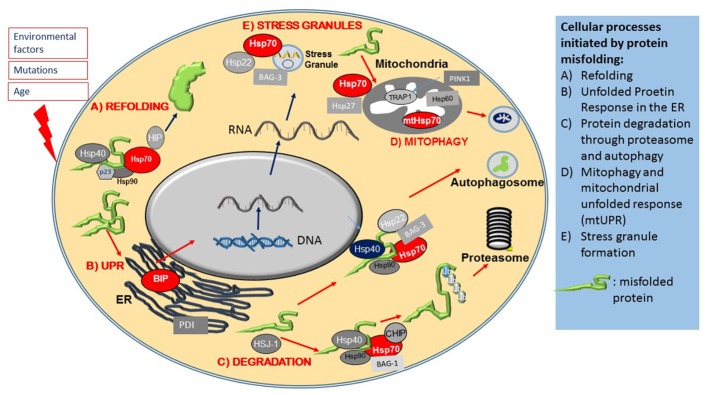
Heat shock proteins and co-chaperones in cellular proteostasis. The Figure schematically summarizes the main roles of members of the Hsp superfamily in distinct steps of protein maintenance. **(A)** Upon mild protein misfolding, Hsp70, in complex with Hsp90 and Hsp40 co-chaperones, facilitate protein refolding through their ATPase activity. **(B)** Protein misfolding is sensed by the ER and initiates the Unfolded Protein Response (UPR), which results in the activation of ER stress proteins including the ER resident Hsps, BIP and PDI. Environmental stresses and disease causing mutations cause the formation of stress granules, which is regulated by Hsp70, Hsp22 and Bag-3. **(C)** Hsp70 and its co-chaperones direct misfolded intracellular proteins to protein degradation pathways. **(D)** Protein misfolding in mitochondria leads to activation of the Mitochondrial Unfolded Response, mediated by specialized mitochondrial Hsps, which together with PINK, monitor mitochondrial integrity and under conditions of mitochondrial dysfunction, initiate the degradation of mitochondria through mitophagy.

The cytosolic chaperone system also functions as a major protein quality control surveillance apparatus, coordinated by cytosolic Hsp70. Upon detection of exposed beta sheets in proteins, Hsp70 and Hsp90 form complex with ATPase activity facilitator co-chaperones such as members of the Hsp40 family and the Hsp90 co-chaperone p23. This large multiprotein complex then transiently binds to misfolded proteins (**Figure 2A**), and through its ATPase activity it renders misfolded proteins into their functionally active confirmation (Mayer and Bukau, 2005). There are studies that suggest that in case protein refolding fails Hsp70 initiates and alternative pathway (Shiber and Ravid, 2014). Thus, in this case Hsp70 recruits a different set of co-chaperones, mainly the E3 ubiquitin ligase CHIP to form the E3 ligase complex that attaches poly ubiquitin chains onto proteins marked for degradation (**Figure 2C**), and also escorts these protein candidates to the proteasome for degradation (Bercovich et al., 1997; Mayer and Bukau, 2005; Kriegenburg et al., 2012; Shiber and Ravid, 2014). The process of physical guidance to the proteasome is aided by an interaction between Hsp70 and another co-chaperone, HSJ1 (DNAJB) a member of the Hsp40 family, whilst docking onto the proteasome requires interaction of Hsp70 with a further co-chaperone, Bcl-2 associated athanogen, BAG-1 and BAG-2 (Lüders et al., 2000; Westhoff et al., 2005).

Larger protein complexes on the other hand, are directed to the autophagy-lysosomal system by Hsp70 complexes associated with the small Hsp, Hsp22 (HspB8) and a co-chaperone, BAG-3 (Crippa et al., 2010; Behl, 2016). A specialized pathway, chaperone mediated autophagy (CMA), involves the recognition of a specific amino acid sequence on client proteins (LysPheGluArgGln) by Hsp70, which directs them to Lamp2A on autophagosomes (Kon and Cuervo, 2010).

The normal maintenance of mitochondria is also dependant of specific subset of Hsps. Mitochondria mostly rely on the import of proteins synthesized in the cytosol and transported to the outer mitochondrial membrane by Hsp70 (Kang et al., 2011). The final folding phase of these proteins involves a specialized mitochondrial chaperone system, consisting of Hsp60 associated with Hsp10, as well as the mitochondrial Hsp70 (mtHsp70) and the mitochondrial Hsp90 analog TRAP1 (Chacinska et al., 2009). Hsp27 (HspB1) is also important in the maintenance of mitochondria and is necessary for normal oxidative phosphorylation and mitophagy (Kang et al., 2011). We have also shown recently that mutations of Hsp27 (HspB1) cause mitochondrial axonal transport deficits and increased ROS production by mitochondria, an effect that is mediated by decreased complex 1 activity (Kalmar et al., 2017).

## The Role of Heat Shock Proteins in the Cellular Stress Response to Increase Stress Resistance

Besides their normal function in cell maintenance, Hsps and the HSR also play a role in protecting calls against harmful environmental stresses (Brown, 1995). The HSR consists of a cascade of events executed by the inducible isoforms of major Hsps as well as co-chaperones, in order to preserve cellular integrity, to mitigate the effects of various environmental stresses and to promote cell survival (Mathew and Morimoto, 1998; Morimoto, 2008). There are various elements of the HSR which protect intracellular compartments in a versatile and stress dependent manner.

### Stress Granule Formation and Disassembly

One early hallmark of stress is the formation of stress granules containing mRNA and proteins that are sequestered into stress granules in order to preserve them and to enable the cell to resume protein synthesis promptly once the cellular stress condition has resolved. Under stress, Hsp70, in complex with other co-chaperones, such as Hsp22 (HspB8) and BAG-3, contributes to stress granule formation (**Figure 2E**) by halting translation. Once the stress has abated, Hsp70 is also necessary for stress granule disassembly (Walters and Parker, 2015; Ganassi et al., 2016).

### Heat Shock Proteins in ER Stress and the Unfolded Protein Response

Another early event in protein misfolding following cell stress is the activation of the ER resident protective UPR pathway. The ER, as a platform for protein synthesis and early modification, is the first intracellular location where proteostasis abnormalities manifest; the UPR is therefore an early stress response mechanism. The UPR is initiated by another Hsp70 family member, BIP (HspA5), which is an ER resident chaperone ATPase (**Figure 2B**). As the amount of misfolded proteins increases, BIP is recruited to chaperone these proteins, which in turn reduces the availability of free BIP that is normally dimerized to ER stress sensors PERK, ATF-6 and IRE1, located in the ER membrane. This enables PERK and IRE1 to activate (through phorphorylation) and initiate the UPR, which then leads to a translational block of other proteins, activation of transcription factors such as XBp1 and NFkappaB, as well as upregulation of UPR target genes, such as the molecular chaperone BIP and the ER chaperone, Protein Disulphate Isomerase (PDI) (Ron et al., 2000; Walters and Parker, 2015; Ruegsegger and Saxena, 2016; Perri et al., 2016). ATF6, after dissociation from BIP, translocates to the Golgi where it is cleaved, resulting in its transcriptionally activated form (Kanekura et al., 2009). Both ATF6 and XBp1 induce expression of ER chaperones such as BIP and the mitochondrial Hsp90 analog GRP94 (Kanekura et al., 2009).

The increased expression of ER chaperones caused by the UPR reduces the load of misfolded proteins and under conditions of moderate stress, resolves the proteostatic stress. However, if ER stress persists, this response is insufficient and persistent activation of PERK leads to the activation of self-destructive apoptotic pathways (Perri et al., 2016; Ruegsegger and Saxena, 2016).

### Mitochondrial Unfolded Protein Response (mtUPR)

Mitochondria also have a protein quality control machinery that senses protein misfolding and other stress conditions within the mitochondrial matrix that damage mitochondria, such as oxidative stress. Under these conditions, mitochondria undergo a mitochondrial unfolded protein response (mtUPR), which also results in the upregulation of mitochondrial Hsps, such as Hsp60, and mitochondrial Hsp70 (HspA9) (Haynes and Ron, 2010) while it also leads to a transcriptional repression of a number of nuclear genes, in order to reduce the load of protein misfolding (Münch and Harper, 2016). It also appears that mitochondria themselves are protective against protein aggregation by actively sequestering non-mitochondrial related, disease causing, aggregation prone proteins, including ALS-causing mutant SOD1 (Liu et al., 2004; Ruan et al., 2017). The import of aggregated proteins into mitochondria is thought to be a compensatory mechanism for a defective protein chaperone system, as reducing cytosolic Hsp70 levels has been shown to increase the uptake of misfolded proteins to mitochondria (Ruan et al., 2017).

Interestingly, the ALS-related proteins FUS and TDP-43, mutations in which are causative for ALS-FTD, have recently been found to interact with chaperones, including mtHsps, and this interaction contributes to mitochondrial dysfunction in a model of ALS (Deng et al., 2015). It has been proposed that mislocalisation of these proteins to mitochondria causes the sequestering of Hsps and impairment of the chaperone system (Bozzo et al., 2016). The mtUPR is coordinated by TRAP1 a mitochondrial Hsp90 analog. Pharmacological inhibition of TRAP1, just like in the case of Hsp90 inhibitors in the cytosol, leads to the initiation of s specific HSR mtUPR, localized to mitochondria (Münch and Harper, 2016). The activation of the mtUPR largely limits cytosolic RNA translation whilst upregulating mitochondrial chaperone expression (Münch and Harper, 2016). TRAP1 is also a target of PINK1, an important regulator of mitochondrial quality control, regulating mitophagy (**Figure 2D**). PINK1 has been shown to associate with TRAP1 and upon oxidative stress, reduces cytochrome release from mitochondria, thereby limiting apoptosis. This effect is abolished in the absence of TRAP1, indicating that TRAP1 is essential for the protective response of mitochondria under conditions of oxidative stress (Pridgeon et al., 2007).

Thus, cellular stress caused by disease-causing mutated proteins or environmental agents, leads to a coordinated HSR that ultimately controls the protein maintenance apparatus: Hsps regulate ER stress, thereby halting protein synthesis, and direct mRNA and proteins to stress granules, whilst also activating the synthesis of cytoprotective chaperones. Hsps, in particular Hsp70, also direct misfolded proteins to either the proteasome or the autophagy apparatus for degradation, or enhance the expression of mitochondrial Hsps to protect against oxidative stress, ultimately aiding cells to return to normal cellular function and to overcome the stress conditions.

## Chaperone Systems as Therapeutic Targets in ALS and Other Protein Misfolding Diseases

Since Hsps undertake such crucial roles in the normal cellular maintenance of proteins, from translation to degradation, as well as the cellular response to stress, they pose an attractive, albeit difficult therapeutic target for protein misfolding disorders such as ALS. It is clear from studies on patient samples and animal models that an age-dependant deficit in cellular Hsp levels is likely to contribute to these largely age-related neurological disorders (Watanabe et al., 2001), while Hsp levels might be elevated outside the CNS in blood (Miyazaki et al., 2016). Indeed, the manifestation of neurodegenerative diseases, including ALS, is largely age-related and correlates with the decline in cellular chaperone systems (Morimoto, 2008; Perez et al., 2012). Neurons are embedded in a network of glial cells, where they metabolically supported. Some types of neurons, like motor neurons, express protective Hsps at a surprisingly low level throughout life and are inherently unable to initiate a rapid HSR in response to stress (Batulan et al., 2003). Thus, instead of engaging a metabolically very active neuronal protein synthetic machinery, there is increasing evidence that surrounding astroglia can supply neurons with vital Hsp proteins which are taken up from the extracellular space (Robinson et al., 2005; Gifondorwa et al., 2007; Taylor et al., 2007; Kalmar and Greensmith, 2009). In ALS, glial cells themselves are known to contribute to disease pathology, and almost all normal glial functions by which they support neurons are impaired (Boillée et al., 2006). Therefore, any Hsp-based therapy will have to engage both neuronal and glial populations.

Several experimental approaches to upregulate Hsp expression in ALS have been undertaken. For example, genetic manipulation that results in overexpression of specific, individual Hsps has been tested in cellular and mouse models of ALS, but with limited success (Bruening et al., 1999; Patel et al., 2005; Krishnan et al., 2008; Sharp et al., 2008). However, considering the complexity of chaperone networks and how the major chaperones (Hsp70 and Hsp90) are dependent on specific co-chaperone systems to exert their diverse intracellular functions, it is perhaps not surprising that manipulation of one single Hsp does not lead to drastic change in disease pathology. Pharmacological approaches to target the upstream transcription factor of the majority of Hsps, HSF-1 have been more successful, as targeting HSF-1 ensures the coordinated synthesis of multiple Hsps, appropriate for the specific type of cell stress. Furthermore, this approach allows for the normal intracellular stoichiometry of Hsps and co-chaperones to be maintained, whilst shifting the level of expression of Hsps overall, within the cell. Such pharmacological approaches include inhibitors of Hsp90, that induces activation of HSF-1, such as GGA (Katsuno et al., 2005) and 17-GAA, a geldanamycin analog (Bonvini et al., 2004; Batulan et al., 2006; Neef et al., 2010), both drugs originally developed for cancer therapy. The Chinese herb Celastrol has also been shown to induce Hsp expression and to be effective at protecting degenerating neurons (Westerheide et al., 2004; Kiaei et al., 2005). However, just upregulating Hsp expression will not always be indicative of cytoprotection, and can simply reflect a harmful activation of the HSR. For example, there is evidence that Celastrol has significant neuronal toxicity and can result inhibition of the proteasome, despite increasing Hsp expression (Kalmar and Greensmith, 2009; Walcott and Heikkila, 2010). A group of hydroxylamine derivatives that act on the stress-sensing of cells and which prolong the activation of HSF-1, have been developed and shown to have significant effects on the survival of neurons in a number of models of neuronal degeneration. For example, Arimoclomol and its derivatives have been shown to have neuroprotective effects in various cellular and *in vivo* models of neurodegeneration, including motor neuron diseases such as ALS and Kennedy’s Disease, injury-induced acute motor and sensory nerve degeneration as well as in retinal (Kalmar et al., 2003, 2008; Kieran et al., 2004; Kalmar and Greensmith, 2009; Malik et al., 2013; Parfitt et al., 2014). The benefits of Arimoclomol are not limited to neurons, as it also restore muscle function and reduce protein aggregation in muscle cells in a model of the muscle disorder Inclusion Body Myositis (Kalmar et al., 2012; Ahmed et al., 2016), another protein-misfolding disorder. Furthermore, a clinical Phase II trial of Arimoclomol in SOD1-ALS patients has recently been completed, and presentation of the results at the 2016 International Symposium on ALS/MND indicated that “the drug was safe and showed ‘trends’ of a beneficial effect. Some people did better on the drug; living longer and with a slower disease progression and decline in respiratory function.” ^1^ Other hydroxylamine analogs, such as BGP-15 have also shown particularly potent effects in preserving muscle function in a model of severe muscular dystrophy (Gehrig et al., 2012), and has protective effects on damaged heart muscle (Gehrig et al., 2012; Sapra et al., 2014).

These results using small molecule compounds, such as BGP-15 and Arimoclomol are therefore encouraging as they provide evidence that it is possible to target the cell’s own defense system to fight disease. However, drug candidates such as Arimoclomol are unlikely to provide a cure for the neurodegenerative conditions, as most of these diseases, by the time diagnosed, are at such an advanced stage, that most of the neurons have already died and are beyond rescue. Thus, early diagnosis will make the therapeutic potential of proteostasis drugs, such as Arimoclomol greater, particularly if applied in a cocktail of other therapeutics targeted at the consequences of protein misfolding, such as antioxidative, antiapoptotic and anti excitotoxic therapies.

## Author Contributions

BK and LG designed the review, BK wrote the first draft.

## Conflict of Interest Statement

The authors declare that the research was conducted in the absence of any commercial or financial relationships that could be construed as a potential conflict of interest.

## Footnotes

1 http://www.alsa.org/news/media/press-releases/trial-of-arimoclomol-120916.html

## Abbreviations

ALS: amyotrophic lateral sclerosis
ALS-FTD: amyotrophic lateral sclerosis and frontotemporal dementia
BAG1: Bcl2-associated athanogene 1
BAG3: Bcl2-associated athanogene 3
BiP: binding immunoglobulin protein
CHIP: C-terminus of Hsp70 Interacting protein
ER: endoplasmic reticulum
ERAD: ER associated degradation
FTD: fronto-temporal dementia
FUS: Fused-in-Sarcoma
Hsp: heat shock protein
HSF-1: heat shock factor 1
HSR: heat shock response
IRE1: Inositol-requiring enzyme 1
IBM: inclusion body myositis
OPTN: optineurin
PDI: protein disulfide isomerase
PERK: protein kinase R (PKR)-like endoplasmic reticulum kinase
PINK1: PTEN-induced putative kinase 1
SOD1: superoxide dismutase 1
SQSTM1: sequestosome-1
TDP-43, TBK-1: tank binding kinase 1
TRAP1: TNF receptor associated protein 1; TAR DNA-binding protein 43
VCP: valosin containing protein
VAP B: vesicle associated protein B
XBP1: X-box binding protein 1
